# Production Cross-Section Measurements for Terbium Radionuclides of Medical Interest Produced in Tantalum Targets Irradiated by 0.3 to 1.7 GeV Protons and Corresponding Thick Target Yield Calculations

**DOI:** 10.3389/fmed.2021.625561

**Published:** 2021-05-12

**Authors:** Charlotte Duchemin, Thomas E. Cocolios, Kristof Dockx, Gregory J. Farooq-Smith, Olaf Felden, Roberto Formento-Cavaier, Ralf Gebel, Ulli Köster, Bernd Neumaier, Bernhard Scholten, Ingo Spahn, Stefan Spellerberg, Maria E. Stamati, Simon Stegemann, Hannelore Verhoeven

**Affiliations:** ^1^Katholieke Universiteit Leuven, Institute for Nuclear and Radiation Physics, Leuven, Belgium; ^2^European Organization for Nuclear Research, European Organization for Nuclear Research, Geneva, Switzerland; ^3^Forschungszentrum Jülich GmbH, Jülich, Germany; ^4^Advanced Accelerator Applications, A Novartis Company, Colleretto Giacosa, Italy; ^5^Insitut Laue-Langevin, Grenoble, France; ^6^Physics Department, University of Ioannina, Ioannina, Greece

**Keywords:** spallation, protons, tantalum, medical applications, terbium, cross-sections

## Abstract

This work presents the production cross-sections of Ce, Tb and Dy radionuclides produced by 300 MeV to 1.7 GeV proton-induced spallation reactions in thin tantalum targets as well as the related Thick Target production Yield (TTY) values and ratios. The motivation is to optimise the production of terbium radionuclides for medical applications and to find out at which energy the purity of the collection by mass separation would be highest. For that purpose, activation experiments were performed using the COSY synchrotron at FZ Jülich utilising the stacked-foils technique and γ spectrometry with high-purity germanium detectors. The Al-27(p,x)Na-24 reaction has been used as monitor reaction. All experimental data have been systematically compared with the existing literature.

## Introduction

Radionuclides are used in medicine as radiopharmaceutical components to target cells and/or follow the metabolism for diagnosis and/or therapeutic purposes. A specific molecule can be linked to different radioisotopes with similar chemical behaviour. In this case, a theranostic approach is possible if two isotopes have properties suitable for either diagnostics or therapy. Since four terbium radioisotopes have properties suitable for medical applications, terbium is regarded as the “Swiss army knife of nuclear medicine” (1). Tb-152 is of interest for imaging through Positron Emission Tomography (PET) and Tb-155 emits γ-rays compatible with the Single Photon Emission Computed Tomography (SPECT) method. Tb-149 has properties suitable for targeted alpha therapy and PET imaging, while Tb-161 is a good candidate for targeted β- therapy and also suitable for SPECT imaging. These radionuclides can be produced e.g., by proton, deuteron or alpha induced reactions on natural or enriched gadolinium (2–7), by neutron irradiation of enriched Gd-160 (8, 9) or spallation reactions on materials like tungsten or tantalum (10–13). The latter production method has been studied in this work in the framework of the ISOLDE facility at CERN, producing radioactive ion beams from 1.4 GeV proton-induced spallation reactions on a solid Ta target. It has been motivated by the observed discrepancies between different experimental cross-section measurements available in the EXFOR data base, especially for Tb-149 and Tb-152. Several collections of Tb-149, Tb-152 and Tb-155 have been carried out at CERN-ISOLDE between 2011 and 2018. Moreover, since 2017, the MEDICIS (Medical Isotopes Collected from ISOLDE) facility focuses on the collection of radionuclides of interest for bio-medical research (14). This installation has already shown the feasibility of providing radionuclides such as Tb-149, Tb-155, Er-169 and Yb-175 for innovative medical research programmes (15). For this purpose, an irradiated target is heated up to high temperatures (above 2,000°C) to allow for the diffusion and effusion of the atoms out of the target to an ion source for subsequent ionisation. The ions are then accelerated and sent through an off-line mass-separator. The radionuclide of interest is mass-separated and subsequently implanted into a support, e.g., a thin metal foil. However, the drawback of this approach is the possible contamination by pseudo-isobars such as, in the case of a Tb-155 collection, the isobaric molecule contaminant Ce-139O-16, which is also implanted into the foil (16). As a consequence, isolation and purification of the Tb-155 collection by radiochemical means is required (17). In the case of a Tb-149 collection, the same issue is observed with a contamination by the Ce-133O-16 molecules (18). With the purpose of optimising the energy to get the highest purity in the Tb isotope production, this article gives cumulative production cross-section data and Thick Target production Yield (TTY) values. It covers the following neutron-deficient radionuclides produced by proton induced spallation of Ta: Tb-149 and its pseudo-isobar Ce-133m, Tb-152, Tb-155, its precursor Dy-155 and its pseudo-isobar Ce-139. It also includes new values in the energy range of interest for the future ISOL@MYRRHA facility at the Belgian Nuclear Research Centre SCK CEN, which will operate at 600 MeV (19).

## Experimental Set-Up and Method

The spallation cross-section data have been experimentally obtained by irradiating thin metallic foils at the COSY accelerator facility at FZ Jülich in Germany. Thin foils of natural tantalum and aluminium were irradiated in the form of stacks with protons of 300 MeV, 500 MeV, 600 MeV, 700 MeV, 900 MeV, 1 GeV, 1.1 GeV, 1.3 GeV and 1.7 GeV. The average proton-beam current ranged from 50 pA up to 300 pA and the irradiation time spanned from 2 up to 5 h for each assembly. Pure tantalum foils of natural isotopic composition (99.988% Ta-181, 0.012% Ta-180) were used as target material to measure the spallation cross-sections of Ta, whereas aluminium foils were used to quantify the average beam current through the Al-27(p,x)Na-24 reaction used as monitor as described in (20) and available from (21). As stated in (20), a maximum uncertainty of 0.20 mb has been applied to the monitor cross section values. It should be noted that these values are not officially endorsed by the IAEA and might be subject to a re-evaluation in the future. For that reason the cross section values used in the calculation are given in Table 1.

**Table 1:** Production cross-section values of the Al-27(p,x)Na-24 reaction used as monitor.

**Energy (MeV)**	**300**	**500**	**600**	**700**	**900**	**1,000**	**1,100**	**1,300**	**1,700**
Na-24 cross section (mb)	9.88 (0.20)	10.53 (0.20)	10.61 (0.20)	10.60 (0.20)	10.41 (0.20)	10.26 (0.20)	10.11 (0.20)	9.78 (0.20)	9.25 (0.20)

*The maximum uncertainty applied to the cross-section values is given in parentheses*.

For each foil the mass was obtained with a precision of ± 0.1 mg. The foils were arranged as stacks of 7 Ta foils (with thicknesses of 2, 6, 10, and 25 μm) and 3 Al foils (thickness of each foil of 50 μm). The first and last Ta foils (10 μm each) only served to equilibrate recoil losses of spallation products and were not analysed by γ-ray spectrometry; the first and last Al foils were discarded as well for the same reason. For each stack, the two Ta foils representing very thin layers of 2 μm (2.7 mg on average) and 6 μm (9.2 mg on average) thickness were measured in a separate study via α-decay spectrometry to retrieve the activity of Tb-149 with an independent method (13). In the present work, the three 25 μm thick foils of Ta (with an average mass of 33.6 mg) and the middle 50 μm thick Al foil (with an average mass of 10.4 mg) of each stack have been analysed by γ-ray spectrometry at different times after the end of irradiation, ranging from 30 min to several days, with a counting time ranging from 1 to 24 h. In addition, the 6 μm thick foils of Ta were measured by γ-ray spectrometry few months after the experiment (13) and analysed to quantify the longest-lived isotopes such as Ce-139. For these purposes single coaxial high-purity germanium detectors were used. The calibration of the detectors has been performed with certified sources of Am-241, Ba-133, Co-57, Co-60, Cs-137, Eu-152, Hg-203, Mn-54, Na-22, Ra-226 and Y-88, leading to a wide range of efficiency values from 60 keV to 1.8 MeV. The sources have been placed and measured at distances of 3, 5, and 10 cm from the detector. The same positions have been used to measure the irradiated foils. The activity values of each produced radionuclide were derived from the recorded γ-ray spectra using the FitzPeaks spectroscopy software (22) as well as half-lives and γ-lines based on the ENSDF database (23). For each radionuclide, all γ-lines with an intensity > 1% and an energy ranging from 60 keV to 1.8 MeV have been considered to build the FitzPeaks library. For the specific cases of Ce-139 and Tb-149 mentioned above, additional activity values were measured in (13) and have been considered in this work via a weighted average. The production cross-section of a radionuclide produced in tantalum σ_Ta_ (see Equation 1) is calculated relative to the cross-section value of the Al-27(p,x)Na-24 reaction σ_Al_ used to monitor the beam current; both quantities are expressed in units of mbarn. This also requires knowledge of the activity of the radionuclide of interest produced in the Ta foils, Act_Ta_ (expressed in Bq) and of the activity of the radionuclide of reference (in our case Na-24) produced in the Al foil, Act_Al_ (expressed in Bq). The mass of the aluminium and tantalum foils, m_Al_ and m_Ta_, respectively, are expressed in grammes while the atomic masses, A_Ta_ and A_Al_, are expressed in g/mol. The production cross-section calculation also takes into account the radioactive decay constant of the radionuclides considered (in second^−1^) as well as the irradiation time (in seconds).

$$\begin{array}{l} \sigma_{\text{Ta}}=\sigma_{\text{Al}}*\frac{\text{Ac}{\text{t}}_{\text{Ta}}*{\text{A}}_{\text{Ta}}*{\text{m}}_{\text{Al}}*\left(1-\exp\left(-\lambda_{\text{Al}}*{\text{t}}_{\text{irr}}\right)\right)}{\text{Ac}{\text{t}}_{\text{Al}}*{\text{A}}_{\text{Al}}*{\text{m}}_{\text{Ta}}*\left(1-\exp\left(-\lambda_{\text{Ta}}*{\text{t}}_{\text{irr}}\right)\right)} \end{array}$$

Equation 1: production cross-section calculation for the radionuclides produced in the Ta foils

The statistical uncertainties on the measured activity in the Ta foils, ΔTa, and in the Al foils, ΔAl, as well as the systematic uncertainty on the monitor cross-section values (13), Δσ_Al_, have been considered in the calculation of the absolute error applying Gaussian error propagation (see values in Table 2). It should be noted that the Ta and Al foils have the same diameter. The beam position as well as his shape was verified before the irradiations through the use of a radiographic film. The aluminium and tantalum foils are considered as being subject to the same primary beam intensity and same beam energy. The latter is justified since the energy loss is <0.5 MeV across each entire stack (24).

**Table 2:** Production cross-section for Ce-133m, Ce-139, Tb-149, Tb-152, Tb-155 and Dy-155, for different proton-irradiation energies between 300 and 1,700 MeV.

	**Energy (MeV)**	**300**	**500**	**600**	**700**	**900**	**1,000**	**1,100**	**1,300**	**1,700**
**Nuclide**	**T_**1/2**_ [23]**	**Production cross-section (mb)**								
Ce-133m	4.9 h *4*	-	-	-	-	0.95^*^	1.70^*^	2.92 (1.08)	4.23 (0.55)	5.03 (0.73)
Ce-139	137.64 d *2*	-	-	2.83 (0.57)	4.26 (0.77)	11.8 (1.9)	-	22.9 (4.0)	28.3 (3.5)	23.0 (2.9)
Tb-149	4.118 h *25*	0.07 (0.02)	4.13 (0.51)	6.62 (0.67)	9.76 (1.06)	10.7 (1.3)	12.4 (1.4)	13.6 (2.0)	13.4 (1.1)	11.4 (1.2)
Tb-152	17.5 h *1*	3.65 (0.53)	19.9 (2.4)	22.5 (2.3)	33.6 (3.8)	40.2 (5.2)	34.1 (3.6)	35.9 (5.6)	34.8 (3.0)	24.4 (2.5)
Tb-155	5.32 d *6*	2.81 (0.89)	25.6 (3.1)	32.5 (3.4)	40.9 (4.7)	49.4 (8.5)	41.2 (5.1)	38.5 (5.9)	41.4 (4.2)	30.1 (3.4)
Dy-155	9.9 h *2*	2.79 (0.36)	25.1 (3.0)	31.2 (3.6)	41.7 (4.8)	37.7 (4.6)	43.5 (5.1)	44.1 (6.8)	41.2 (4.1)	29.8 (3.4)

* *upper limits calculated using minimum detectable activity (MDA) values extracted with FitzPeaks (22)*.

*Absolute uncertainties including statistical and systematic errors are enclosed with the parentheses*.

## Results and Discussions

### Cross-Sections

The cross-section results for Ce-133m, Ce-139, Tb-149, Tb-152, Tb-155, and Dy-155 are presented and discussed in this section. All numerical values are summarised in Table 2.

#### Ce-133m

**Figure 1:**
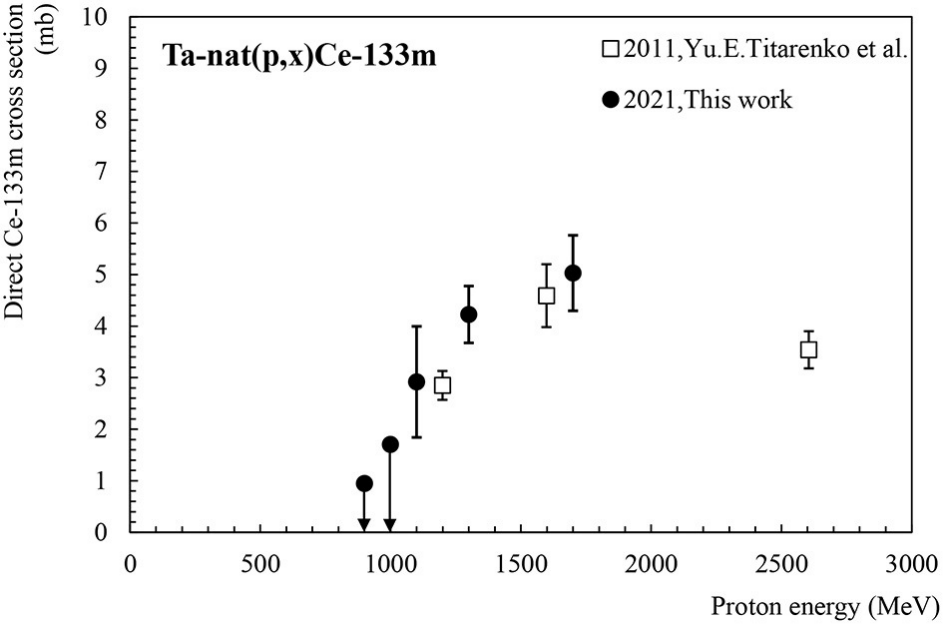
Independent cross-section for Ce-133m production as a function of the proton beam energy.

Ce-133m has a half-life of 4.9 h and its main detectable γ-line is observed at 477.2 keV (I = 39%). There is no known feeding by precursor decays and thus, data in Table 2; Figure 1 can be regarded as the independent production cross-section for Ce-133m. The only data set available in the literature is the one published in 2011 by Titarenko et al. (12) showing a very good agreement with the additional points contributed by this work. No activity could be detected in the foils irradiated at an energy lower than 1.1 GeV since the produced activities were below the minimum detectable activity (MDA). These MDA values extracted from the FitzPeaks software (22) were used to give an upper limit estimate of the production cross-section at 900 MeV and 1 GeV. The corresponding values are plotted as arrows pointing downwards in Figure 1. The Ta-nat(p,x)Ce-133m excitation function shows a maximum at 1.7 GeV with 5 mb.

#### Ce-139

**Figure 2:**
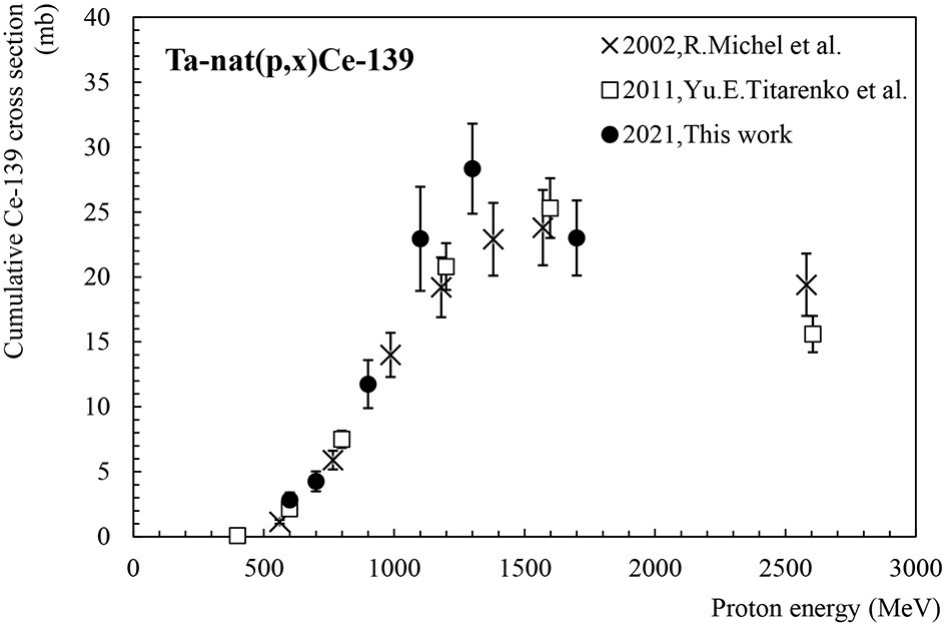
Cumulative cross-section for Ce-139 production as a function of the proton beam energy.

Figure 2 presents the cumulative production cross-section of Ce-139. Ce-139 has a half-life of 137.64 days and decays to La-139 (stable) by emitting an intense γ-ray at 165.9 keV (I = 80%). Its activity has been deduced from γ-ray spectrometry measurements performed several days and several months after the end of irradiation to ensure the decay of Pr-139 (T_1/2_ = 4.4 h) and its precursors into Ce-139 (13). Two data sets are available in the literature for the cumulative production of Ce-139 in tantalum (11, 12). Our new cumulative production cross-section data presented in Figure 2 generally show good agreement with the trend indicated by the values obtained by Michel et al. (11) and by Titarenko et al. (12), considering the reported errors. Yet, our points at 1,100 and 1,300 MeV are about 20% higher than the overall tendency. Ce-139O-16 is an isobaric molecule which during the mass separation step is collected at the same time as Tb-155, generating a radioactive impurity in the final product.

#### Tb-149

**Figure 3:**
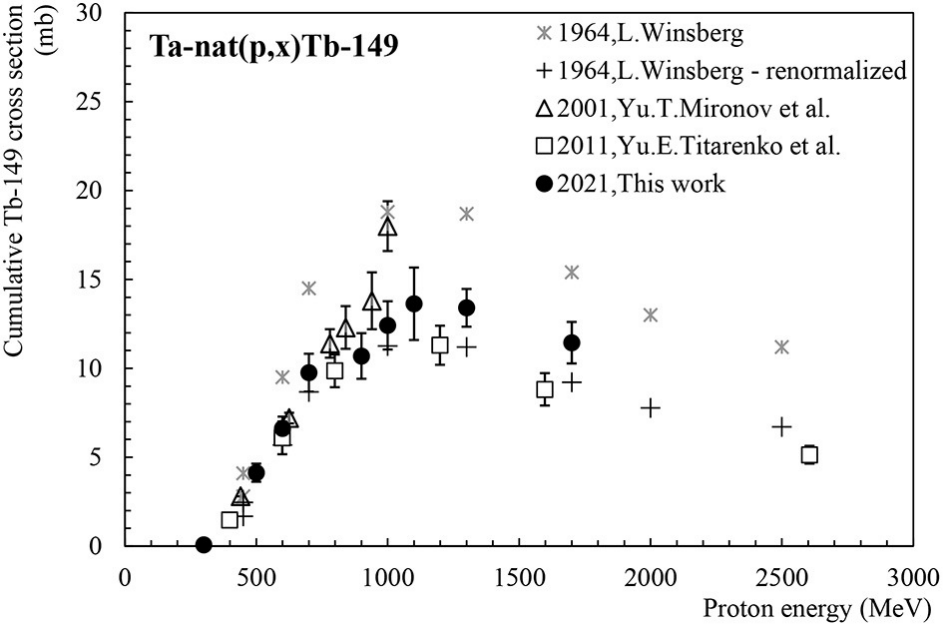
Cumulative cross-section for Tb-149 production as a function of the proton beam energy.

Tb-149, with a half-life of 4.1 h, is an α-emitter of high interest for α-therapy (low α-energy at 3.97 MeV) which has also sufficient positron emission for PET imaging (mean β+ energy of 730 keV and total β+ intensity of 7%). The feasibility of performing PET images with this radionuclide, produced at CERN-ISOLDE and labelled with a biomolecule, has been successfully demonstrated (25). Figure 3 presents the cumulative cross-section for the production of Tb-149 by proton-induced spallation reactions in tantalum. This radionuclide is produced directly and through the decay of the mother radionuclide Dy-149 that has 4 min half-life and thus its majori will have decayed into Tb-149 only a few hours after the end of an irradiation. Three data sets are available in the literature for the production cross-section of Tb-149 from proton induced spallation reactions in Ta. Considering the general tendency of the existing data, the values published by Winsberg (26) generally overestimate the cross-section. It has to be noted that the author states that the activities were retrieved by α-spectrometry. In the original publication one reads that “the branching ratio for alpha decay of the ground state is approximately 10%” (26). According to the latest evaluations, the branching ratio is currently known to be 16.7%, as can be seen in (23) as well as in (27). As a consequence, Winsberg's cross-section values should be re-evaluated. After applying a correction factor of 1.67 to Winsberg's values, one can conclude that our results are in good agreement with all available data sets (12, 26, 28).

#### Tb-152

**Figure 4:**
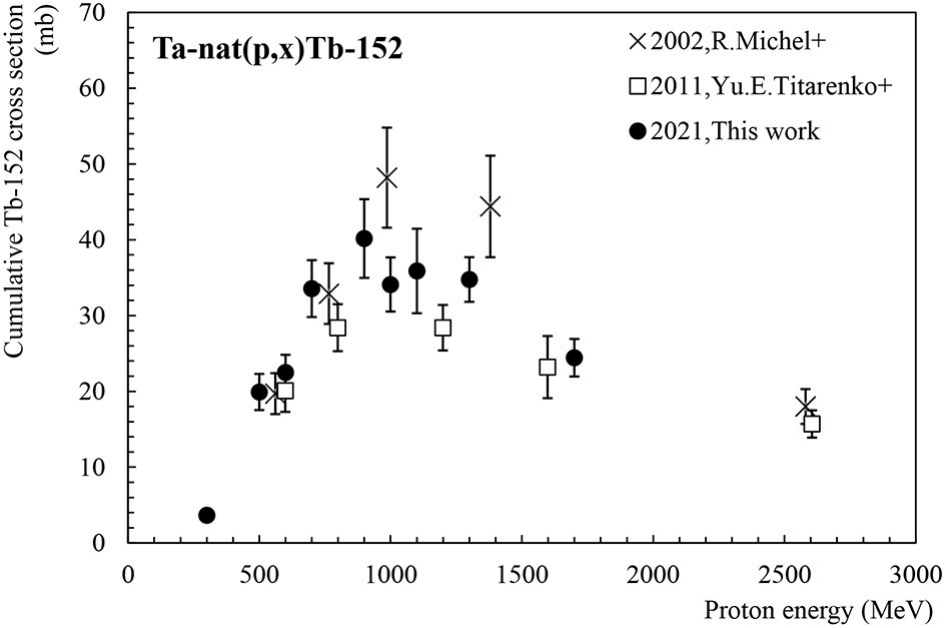
Cumulative cross-section for Tb-152 production as a function of the proton beam energy.

Tb-152 (T_1/2_ = 17.5 h) is a radionuclide useful for PET imaging. It has already been used for first-in-human demonstrations with DOTATOC and PSMA-617 radiopharmaceuticals, respectively, from mass-separated Tb-152 provided by CERN-ISOLDE (29, 30). These studies have shown at late time points improved diagnostic quality with respect to Ga-68. Tb-152 can be directly produced by spallation but is also fed by internal transition (with 78.8% branching ratio) of its metastable state Tb-152m (T_1/2_ = 4.2 m) and the decay of Dy-152 (T_1/2_ = 2.4 h). Figure 4 shows the cumulative production cross-section of Tb-152. Data sets available in the literature (11, 12) are compared with our new values. Differences can be observed between the values obtained by Michel et al. (11) and the data measured by Titarenko el al. (12), in the energy range going from 1 to 1.5 GeV. Our data lie between both data sets and are consistent with the previous values considering the uncertainties. The maximum of the cross-section is estimated to be located at 1 GeV with a cross-section value of 40 mb.

#### Tb-155

**Figure 5:**
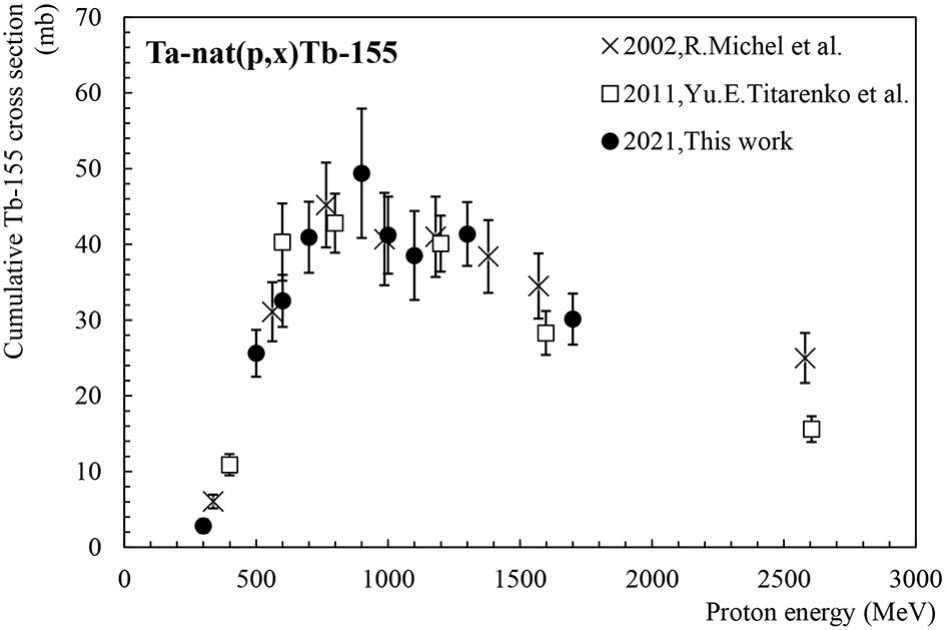
Cumulative cross-section for Tb-155 production as a function of the proton beam energy.

**Figure 6:**
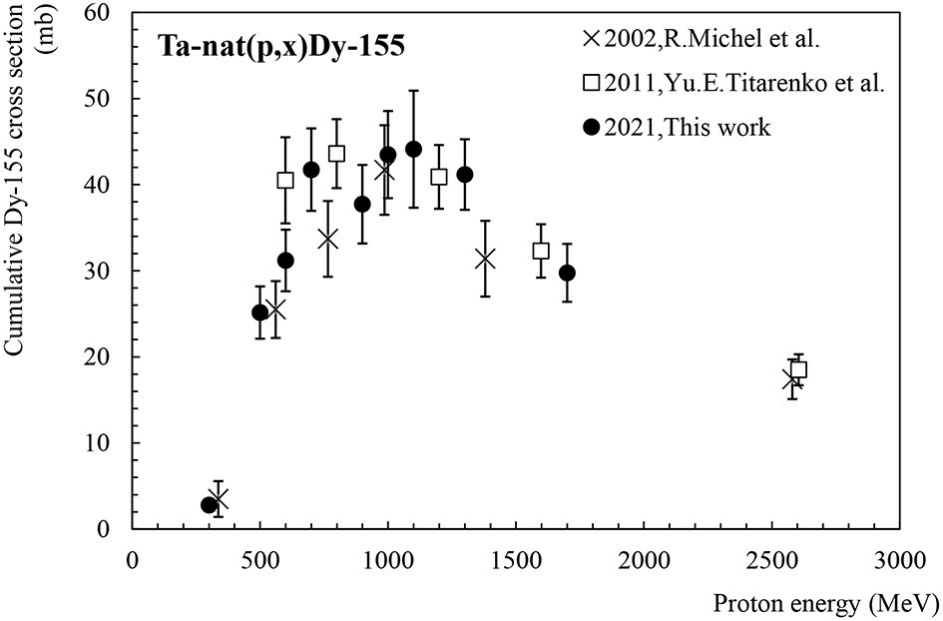
Cumulative cross-section for Dy-155 production as a function of the proton beam energy.

Tb-155 (T_1/2_ = 5.3 days) is a longer-lived terbium isotope suitable for SPECT imaging (16). It decays into stable Gd-155 by emitting four main γ rays at 86.5 keV (I = 32.0%), 105.3 keV (I = 25%), 180.1 keV (I = 7.45%) and 262.3 keV (I = 5.29%) which are easily detectable by γ-ray spectroscopy. Figure 5 shows the Tb-155 cumulative production cross-section, measured after the decay of Dy-155 whose production cross-section is shown in Figure 6. Our new data set is in very good agreement with the existing data published by Michel et al. (11) and Titarenko et al. (12). The maximum of the cross-section is located at 900 MeV with 45 mb.

#### Dy-155

Figure 6 shows the cumulative production cross-section for Dy-155. Dy-155 has a half-life of 9.9 h and decays to Tb-155 (T_1/2_ = 5.3 days) by electron capture and β+ emission, also emitting an intense main γ-ray at 226.9 keV (I = 68.4%). Dy-155 may also be used as a precursor to Tb-155 in on-line conditions where its extraction efficiency is superior to that of Tb-155 (16). Two data sets are available in the literature for the production of Dy-155 via proton-induced spallation of tantalum (11, 12). As can be seen, our new data set is in very good agreement with the existing data, within error bars. The maximum of the cross-section values is located around 1 GeV with 45 mb, similarly to the Tb-155 cross-section.

#### The Difference: σ_Cum._Tb-155–σ_Cum._Dy-155

**Figure 7:**
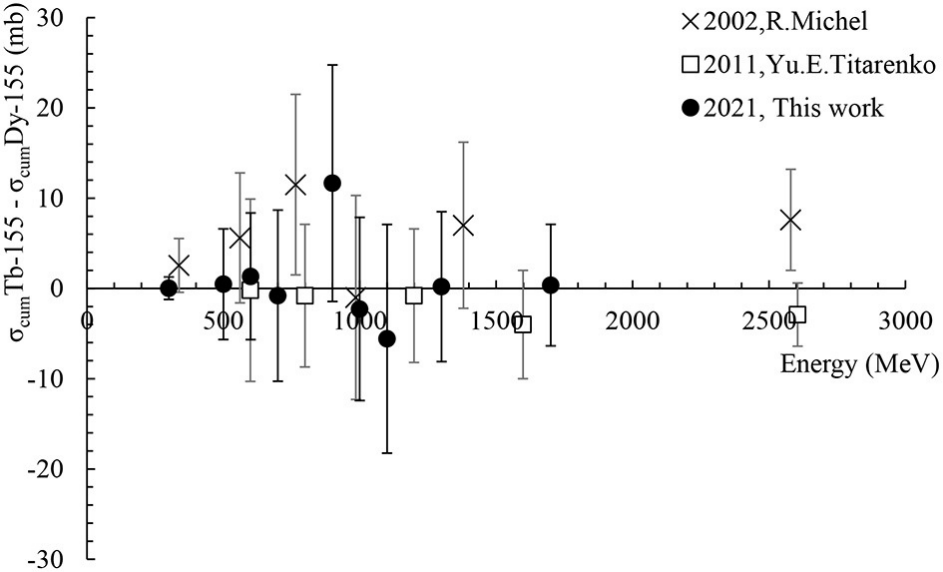
Difference [σ_Cum._Tb-155–σ_Cum._Dy-155] based on the cross-section data measured in “This work (2021)” and the ones available in the literature.

The previously presented cumulative cross-sections σ_Cum._Tb-155 and σ_Cum._Dy-155 show rather similar values over the entire energy range, the former being dominantly created by decay of the latter. In an attempt to illustrate the individual cross-section of Tb-155 without the contribution from the decay of Dy-155, Figure 7 depicts the difference [σ_cum_Tb-155–σ_cum_Dy-155] performed between the available experimental results. While the individual production cross-section of Tb-155 could also be deduced by correcting for the contribution originating from the Dy-155 decay, it requires access to the raw experimental activities. As these are not available for the reference values from literature, the authors chose to illustrate the direct Tb-155 production via the aforementioned difference of the cumulative cross-sections. In the case of our measurements the direct production cross-section has nevertheless been derived via the Dy-155 decay as well and results consistent with Figure 7 have been found. It should be pointed out that negative values in the graph are artefacts due to the subtraction of measurement results that are numerically very close with statistical and systematic errors larger than the differences between the two data sets. Although this result can't be taken as an individual cross section, the convergence of the differences towards 0 confirms that the direct production probability of Tb-155 is minor. Therefore, for an online^1^ collection process as it is performed at ISOLDE, this motivates the exploitation of indirect production via the decay of Dy-155.

### Thick Target Production Yields (TTYs)

The equation to be solved in order to calculate a thick target production yield (31) is shown in Equation 2.

$$\begin{array}{l} \text{ACT}\left(\text{E}\right)=\varphi \cdot \frac{N_{A}.\rho }{\text{A}}\cdot \left(1-\text{exp}\left(-\lambda \cdot {\text{t}}_{\text{irr}}\right)\right)\cdot \int_{\text{Emin}}^{Emax}\frac{\sigma \left(\text{E}\right)}{\chi }\cdot \text{dE} \end{array}$$

Equation 2: Thick Target production Yield formula

The thick target yield values are normalised per μAh. Therefore, φ is the number of particles in 1 μA. The irradiation time t_irr_ is set to 1 h. It implies an integral calculation over a defined energy range, E_min_-E_max_, on the available production cross section data σ(E) and on the deposited energy χ = dE/dx. One has to keep in mind that the formalism of Equation 2 and the results presented hereafter, do not account for the production of radionuclides due to ensuing secondary particle showers. In cases where production originating from secondaries become of importance a full Monte Carlo simulation would have to be carried out.

In this work, cubic smoothing-spline fits have been performed using the Scientific Python module with the cross-section data, utilising variable smoothing factors and taking into account the uncertainties of the data. The fits have been performed on the cross-section values extracted from “This work (2021)” as well as on all the data available in the literature including our new data set referred to as “All data” in the following figures. Additional information can be found in the Supplementary Materials. In addition, energy loss calculations have been performed using the SRIM software (24) and FLUKA simulations (32, 33). FLUKA is of particular interest for our study with proton energies exceeding 300 MeV, as it allows to take energy loss effects due to hadronic interactions into account. Respective simulations have been carried out between 1 MeV and 2.7 GeV considering thin tantalum foils with a density ρ of 16.6 g/cm^3^, which is the nominal density of tantalum used as target material during the COSY experiment. Using this energy range and similar target characteristics also the SRIM software has been used for evaluating the energy loss. Table 3 gives the ratios between the dE/dx values calculated by FLUKA (statistical uncertainties <0.9%) and the values obtained with the SRIM software, for some specific beam energies.

**Table 3:** Comparison among the stopping power (dE/dx) calculated with SRIM and FLUKA.

**Energy** **(MeV)**	**1**	**50**	**100**	**300**	**500**	**600**	**1,000**	**1,400**	**1,500**	**2,000**	**2,500**
Ratio dE/dx FLUKA/SRIM	1.0	1.0	1.0	1.1	1.1	1.2	1.3	1.4	1.5	1.6	1.6

**Figure 8:**
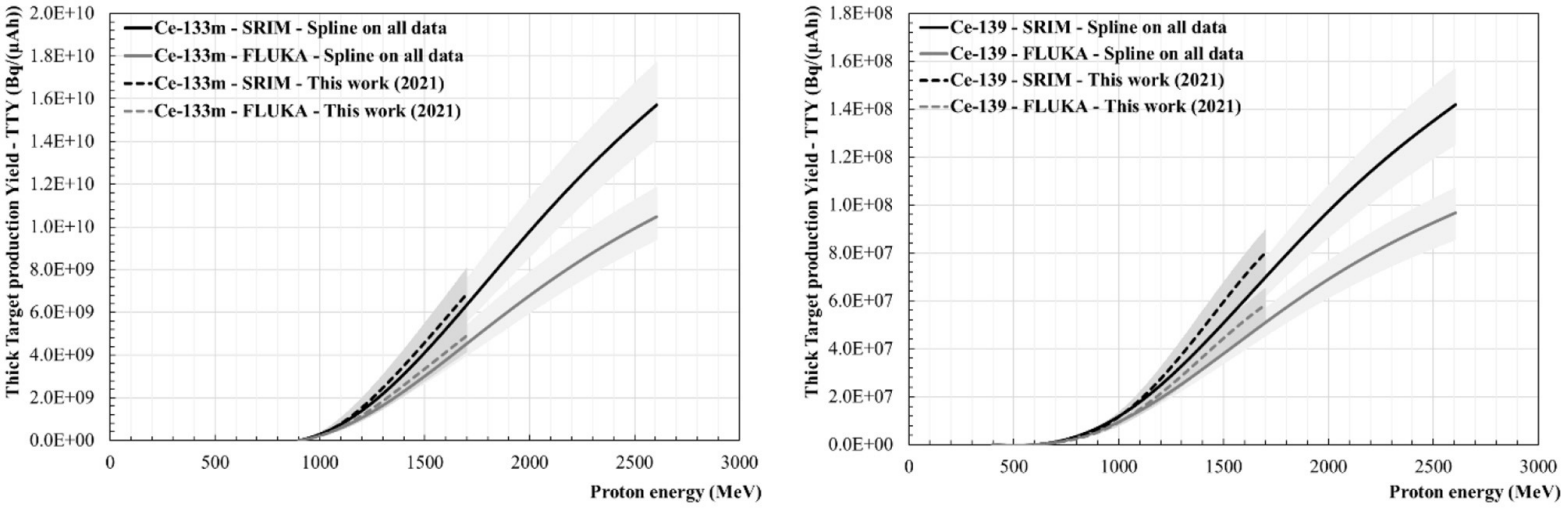
Thick Target production Yields (TTY) for Ce-133m **(Left)** and for Ce-139 **(Right)**.

**Figure 9:**
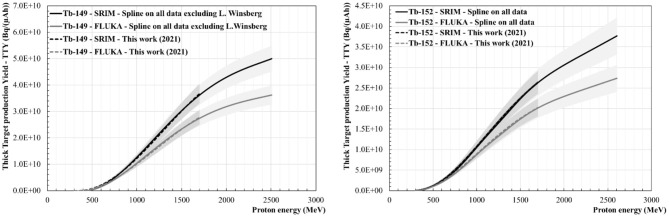
Thick Target production Yields (TTY) for Tb-149 **(Left)** and for Tb-152 **(Right)**.

**Figure 10:**
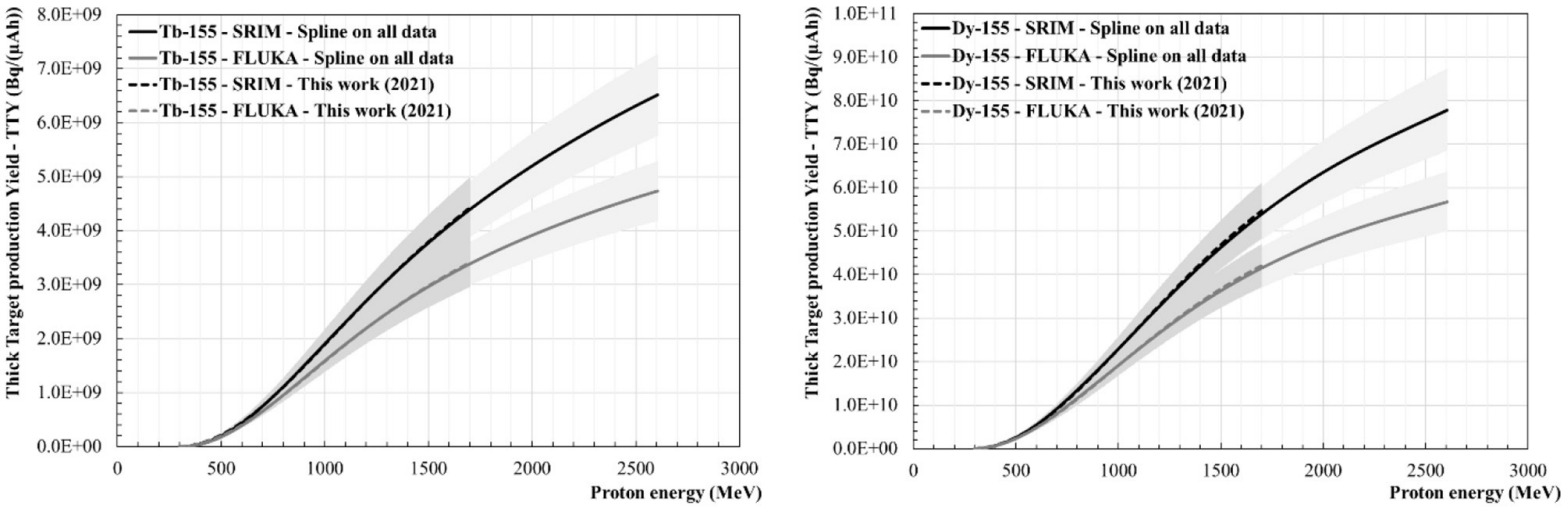
Thick Target _production_ Yields (TTY) for Tb-155 **(Left)** and for Dy-155 **(Right)**.

Figures 8–10 show the Thick Target production Yields for Ce-133m, Ce-139, Tb-149, Tb-152, Tb-155 and Dy-155. There are four scenarios which have been studied. The black lines stand for a TTY calculation performed using dE/dx given by the SRIM software, whereas the grey lines show the results from dE/dx values computed using FLUKA. The full lines use the spline fit performed on all the data found in the literature. The dashed lines give results based on the new production cross section data set calculated in “This work (2021)”. The uncertainties are shown in the graphs as grey shaded areas. They have been calculated by evaluating the minimal and maximal cubic smoothing-splines from the cross-section data. The same colour and pattern scheme have been applied to the following figures. Depending on the radionuclide, a maximum difference ranging from 27 to 33% can be seen in the case of the TTY calculations performed using SRIM in comparison with FLUKA, the latter giving higher dE/dx values for E > 100 MeV. For all radionuclides there is good agreement between the TTY calculated based on the cross-section values measured in “This work (2021)” and based on all the data sets available in the literature.

#### Ce-133m and Ce-139 Thick Target Production Yields

Figure 8 shows the thick target production yields calculated for Ce-133m and Ce-139. Among the four scenarios discussed before, the Ce-133m TTY values at 1.4 GeV (CERN-ISOLDE/MEDICIS energy) range from 2.3 GBq/(μAh) ± 24% to 3.5 GBq/(μAh) ± 22%.

At 600 MeV (ISOL@MYRRHA energy), the Ce-139 TTY values are found to be between 38 and 43 kBq/(μAh), with an uncertainty of ± 29%. At 1.4 GeV this value is between 32 and 49 MBq/(μAh) with an uncertainty of ± 15%.

#### Tb-149 and Tb-152 Thick Target Production Yields

Figure 9 shows the thick target production yields calculated for Tb-149 (left graph) and Tb-152 (right graph). At 600 MeV, the Tb-149 TTY values range from 1.5 to 2.1 GBq/(μAh) (with an uncertainty of ± 13%) and at 1.4 GeV are between 21 and 27 GBq/(μAh) (± 10%), which represents a difference larger than a factor of 10. The Tb-152 TTY calculations show very good agreement between the fit performed on our new cross-section data set and the one performed taking into account all cross-section data available in the literature. At 600 MeV a TTY value between 2.0 and 2.5 GBq/(μAh) can be derived with an uncertainty of 10 and 15%, respectively. This value is ranging from 16 to 20 GBq/(μAh) at 1.4 GeV with an uncertainty of ± 12%.

#### Tb-155 and Dy-155 Thick Target Production Yields

Figure 10 shows the thick target production yields calculated for Tb-155 and its precursor Dy-155. It should be noted that the Tb-155 TTY is coming from experimental data measured after the complete decay of Dy-155. Figure 10 shows that for both radionuclides there is very good agreement of the TTY values obtained from the spline fit performed on our data set only and from the one including all data sets. The difference between the values calculated using FLUKA and using SRIM is up to 27% for both radionuclides. At 600 MeV the Tb-155 TTY value ranges from 390 MBq/(μAh) (±12%) to 450 MBq/(μAh) (±15%) whereas, for the same energy, the Dy-155 TTY values range from 4.9 to 5.4 GBq/(μAh) with a relative uncertainty of 15%, which would become 300 to 340 MBq/(μAh) (±15%) of Tb-155 after 40 h. At 1.4 GeV, the Tb-155 TTY values are between 2.7 GBq/(μAh) (±12%) and 3.5 GBq/(μAh) (±13%). The Dy-155 activity values are more than 10 times higher and range from 33 to 42 GBq/(μAh) (±12%), which would scale to between 2.1 and 2.6 GBq/(μAh) (±12%) of Tb-155 after 40 h.

### Thick Target Yield Ratios and Assessment of Tb-149, Tb-155 and Dy-155 Purity

Tb-149 and Dy-155/Tb-155 collections can be contaminated by their pseudo-isobaric oxide forms, Ce-133O-16 and Ce-139O-16, respectively, when collecting these radionuclides through mass separation. This section presents purity levels expressed in terms of activity which are based on the in-target production TTY values (in Bq/μAh) presented in section Thick Target Production Yields (TTYs) and on Ce isotopes as only isobaric contaminants of Tb-149, Tb-155 and Dy-155. The other collected isobars will either decay into the radionuclide of interest (e.g., Ho-155 decaying into Dy-155, decaying itself to Tb-155) or be a decay product of the collected isotope (e.g., Gd-155 from Tb-155 decay). In both cases, isobars can be chemically separated after the mass separation and the collection. It should be noted that these in-target TTY ratios are not equal to those of the actually collected samples at it is done at ISOLDE/MEDICIS, since the diffusion, effusion and ionisation efficiencies have to be considered, which differ for each element and isotope. Efficiency values of the order of 1% have been achieved with Tb at CERN-MEDICIS in 2018 (15) and further developments have been carried out to increase this value up to 10% in 2019 and 2020 (34).

#### Tb-149 Purity

**Figure 11:**
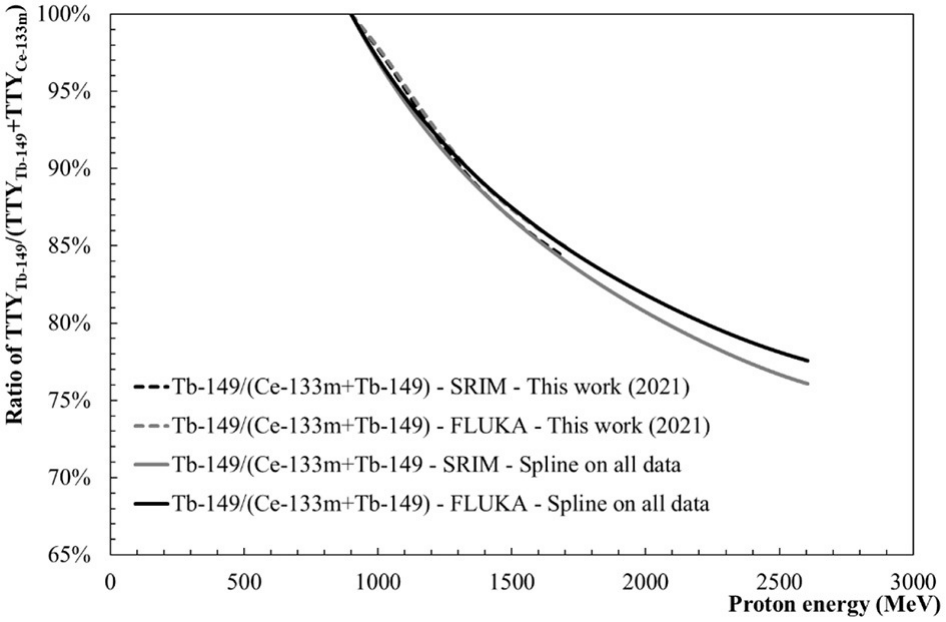
Tb-149 purity with the ratio TTY_Tb−149_/(TTY_Ce−133m_+TTY_Tb−149_).

Figure 11 allows for assessing the purity of Tb-149 calculated as the TTY ratio between Tb-149 and Ce-133mO-16 production: TTY_Tb−149_/(TTY_Ce−133m_+TTY_Tb−149_). Slightly lower values are calculated in the case of the spline fit performed on the data set from “This work (2021)” in comparison with the values obtained when considering all data sets. The extracted activity ratios show that considering only Ce-133m as in-target contaminant for the collection of Tb-149, a purity higher than 99% can be reached if the proton beam energy is below 940 MeV. Keeping the beam energy below 900 MeV allows for achieving a purity higher than 99.9%. At 1.4 GeV ratios between 88 and 89% are expected. It has to be noted that Ce-133m and Tb-149 have similar half-lives with 4.9 and 4.1 h, respectively. Therefore, the purity levels shown in Figure 11 are representative of the activity purity of the final product that will not vary substantially as a function of time.

#### Tb-155 and Dy-155 Purity: Considerations on Offline and Online Mass Separation

**Figure 12:**
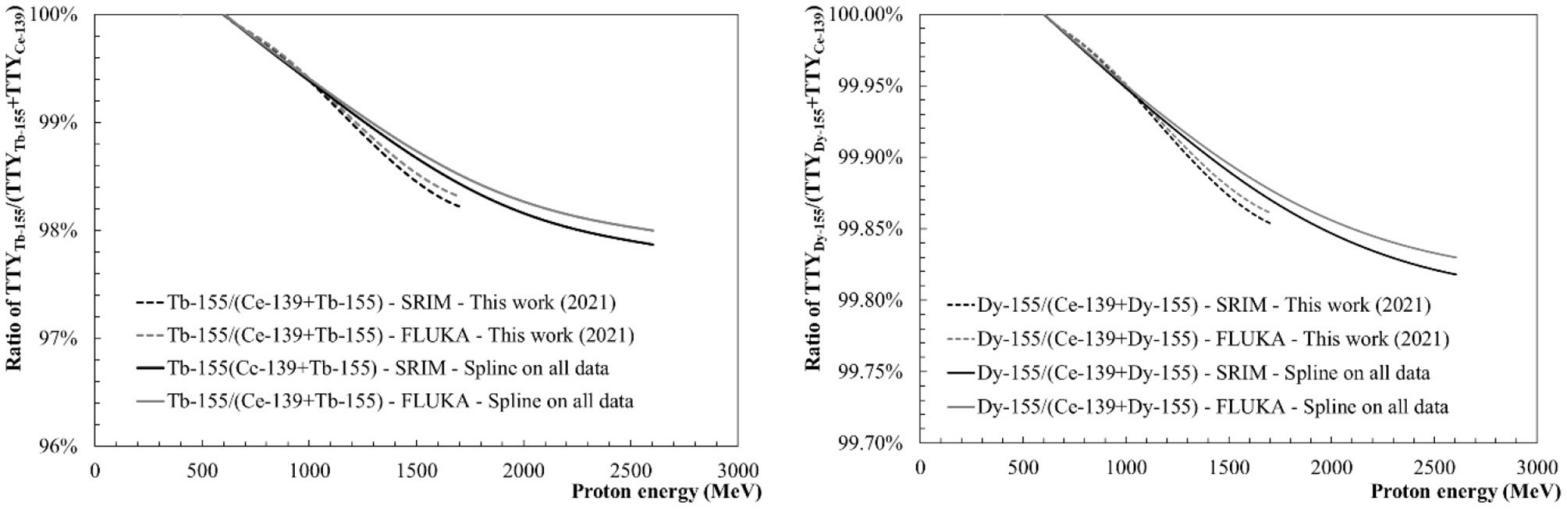
Tb-155 purity with the ratio TTY_Tb−155_/(TTY_Ce−139_+TTY_Tb−155_) **(Left)**–Dy-155 purity with the ratio TTY_Dy−155_/(TTY_Ce−139_+TTY_Dy−155_) **(Right)**.

Figure 12 shows the Tb-155 (left) and Dy-155 (right) activity purity levels, for which the collection by mass separation can be affected by the presence of Ce-139O-16 molecules. Figure 12 (left) shows that a Tb-155 purity higher than 99% can be reached if the beam proton energy is below 1,200 MeV. With a beam energy below 660 MeV a purity higher than 99.9% can be reached. At 600 MeV and 1.4 GeV the purity level would reach >99.9 and 98.6% respectively. Figure 12 (right) shows that a Dy-155 purity higher than 99.9% can be achieved with a proton beam impinging a tantalum target with an energy below 1.4 GeV. With an energy above 1.4 GeV and up to 2.6 GeV Dy-155 is produced in the target with a purity higher than 99.8%, once again considering Ce-139 as the only contaminant of Dy-155 in the target.

However, it should be noted that, in practise, an offline collection (i.e., offline refers to a collection performed after the target has been irradiated) of Tb-155, as performed at CERN-MEDICIS, starts few days after the end of irradiation to allow for the decay of part of the Dy-155 nuclei into Tb-155 nuclei in the target. Therefore, one has to keep in mind that the Dy-155/Tb-155 ratio will evolve with time. The Dy-155 activity will be considerably higher than the Tb-155 activity at the end of irradiation. Then, 40 h after the end of irradiation, both radionuclides will show similar activities which lead to a radionuclidic purity close to 0.5. After this period the relative proportion of Dy-155, being the main impurity in the sample, decreases while continuously feeding Tb-155 through its decay. A decay time above 40 h will reduce the Tb-155 activity but will significantly increase its isobaric purity.

Online collections of Dy-155, producing Tb-155 by decay, have been already performed at CERN-ISOLDE (16) from a Ta target irradiated with a 1.4 GeV proton beam. After a Dy-155 collection performed in 2013 at CERN-ISOLDE, a sample has been successfully shipped and processed at the Paul Scherrer Institute in Switzerland. Three days after the end of the collection, the Dy-155 activity was 280 kBq, the Tb-155 activity was 136 MBq and the Ce-139 activity was 4.7 MBq (16). This results in a Tb-155 activity purity of 96.5% 3 days after the end of collection. It also gives a Dy-155 purity level of 99.99% at the end of the collection, which corresponds to an amount of Ce-139 impurities 10 times lower than estimated above using the in-target TTY values. Yet, one has to take into account the corresponding diffusion, effusion and ionisation efficiencies of Dy-155 and Ce-139O-16 and the additional efficiency of the CeO molecular formation.

One may conclude that collections of Tb-155 with ion ratios as observed here will invariably require a chemical post-separation to assure sufficient radionuclidic purity for clinical use. The issue of Dy-155 as a contaminant of Tb-155 could be resolved by a decay time much longer than 40 h [e.g., 3 days as done in (16)] but at the expense of a rising contribution of the Ce-139 activity.

Table 4 summarises the previously discussed TTY values expressed in GBq/μAh, as well as the purity levels based on Ce-133m and Ce-139 as isobaric contaminants, at 600 MeV and 1.4 GeV.

**Table 4:** TTY values for Tb-149, Tb-152, Tb-155 and Dy-155 (in GBq/μAh) and purity levels of Tb-149, Tb-155 and Dy-155 based on Ce-133m and Ce-139 as isobaric contaminants.

	**Thick Target production Yield (GBq/μAh)**				**Purity levels based on Ce-133m and Ce-139 as isobaric contaminants**		
	**Tb-149**	**Tb-152**	**Tb-155**	**Dy-155**	**Tb-149**	**Tb-155**	**Dy-155**
600 MeV	1.5–2.1	2.0–2.5	0.39–0.45	4.9–5.4	100%	> 99.9%	> 99.9%
1.4 GeV	21–27	16–20	2.7–3.5	33–42	88–89%	98.6%	> 99.8%

## Conclusions

Spallation cross-sections have been measured at the COSY synchrotron at FZ Jülich with fixed energies between 0.3 and 1.7 GeV. This article focuses on the production of three terbium radioisotopes of medical interest Tb-149, Tb-152 and Tb-155 as well as on Dy-155, which feeds Tb-155 by decay, and Ce-133 and Ce-139, which are collected by mass separation as molecular isobaric radioactive contaminants. Some discrepancies between the existing data sets could be highlighted but an overall good agreement has been found between our new data set and the ones available in the literature. In the light of more recent findings for branching ratios, an official re-evaluation of Winsberg's cross-section values could be of interest. Thick Targets production Yield (TTY) values and ratios have been calculated at different energies, using our new experimental cross-section data set as well as the ones available in the literature. These calculations have been carried out using the different computational models of SRIM and FLUKA to determine the energy deposition. One sees the onset of hadronic effects which become more important with increasing energies above 100 MeV. Depending on the energy, neglecting these effects can lead to an overestimation of the calculated TTY. Using their corresponding TTY, activity purity levels of the Tb-149, Tb-155 and Dy-155 radionuclides have been assessed, considering their pseudo-isobaric molecules as sole contaminant. The production of these radionuclides of medical interest via spallation reactions in tantalum is now better known and it will allow for optimising their production at proton energies available at ISOLDE and MEDICIS at CERN (Switzerland), but also at ISAC or ARIEL at TRIUMF (Canada) as well as at the future ISOL@MYRRHA facility at the Belgian Nuclear Research Centre SCK CEN and other high energy proton accelerators worldwide. While this article focusses on terbium isotopes for medical applications, the complete analysis of all radionuclides quantified from the measured γ-spectra will be discussed in a forthcoming article.

## Data Availability Statement

The original contributions presented in the study are included in the article/Supplementary Material, further inquiries can be directed to the corresponding author/s.

## Author Contributions

All authors listed have made a substantial, direct and intellectual contribution to the work, and approved it for publication.

## Conflict of Interest

RF-C was employed by AdAcAp during his PhD thesis under the MEDICIS-promed agreement, when the experiments presented in this work have been conducted. The research has been perform within the framework of the MEDICIS-promed Horizon 2020 Marie Sklodowska-Curie Innovative Training Network. OF, RG, BN, BS, IS, and SS were employed by Forschungszentrum Julich GmbH when the experiments presented in this work have been conducted. The remaining authors declare that the research was conducted in the absence of any commercial or financial relationships that could be construed as a potential conflict of interest.

## Footnotes

^1^online refers to a collection performed at the same time as the target irradiation, as is done at CERN-ISOLDE.
