# Supplementary material for: Production Cross-Section Measurements for Terbium Radionuclides of Medical Interest Produced in Tantalum Targets Irradiated by 0.3 to 1.7 GeV Protons and Corresponding Thick Target Yield Calculations
Source: Front Med (Lausanne). 2021 May 12;8:625561. doi: 10.3389/fmed.2021.625561 (PMC8149945; doi:10.3389/fmed.2021.625561)
Supplement: Supplementary file 1 [file Data_Sheet_1.docx]

Supplementary Material

## Supplementary Figures

**Supplementary Figure 1**: Independent cross-section for the production of Ce-133m and associated cubic-splines.

**Supplementary Figure 2**: Cumulative cross-section for the production of Ce-139 and associated cubic-splines.

**Supplementary Figure 3**: Cumulative cross-section for the production of Tb-149 and associated cubic-splines.

**Supplementary Figure 4**: Cumulative cross-section for the production of Tb-152 and associated cubic-splines.

**Supplementary Figure 5**: Cumulative cross-section for the production of Tb-155 and associated cubic-splines.

**Supplementary Figure 6**: Cumulative cross-section for the production of Dy-155 and associated cubic-splines.
